# Prognostic role of PD-L1 for HCC patients after potentially curative resection: a meta-analysis

**DOI:** 10.1186/s12935-019-0738-9

**Published:** 2019-01-29

**Authors:** Gao-Min Liu, Xu-Gang Li, Yao-Min Zhang

**Affiliations:** The 2nd Department of Hepatobiliary Surgery, Meizhou People’s Hospital, No. 34 Huangtang Road, Guangdong, 514031 China

**Keywords:** PD-L1, HCC, Prognosis, Clinicopathological features

## Abstract

**Background:**

A series of studies has investigated the prognostic role and clinical significance of programmed death ligand 1 (PD-L1) in hepatocellular carcinoma (HCC). However, the results were inconsistent. We aimed to clarify the prognostic role of PD-L1 and relationship between PD-L1 expression and several important clinicopathological features.

**Methods:**

PubMed, EMBASE and the Science Citation Index Expanded were systematically searched. All cohort or case–control studies comparing the prognosis and clinical features between the high PD-L1 and low PD-L1 groups were included. Publication bias was evaluated using funnel plots and Begg’s test. Subgroup analysis, sensitivity analysis and meta-regression analysis were performed.

**Results:**

Seventeen studies including 2979 patients were eligible. The overall survival (OS) was not significantly different between the high and low PD-L1 groups (hazard ratio [HR]: 1.27; 95% confidence interval [CI] 0.98–1.65: P = 0.07) with significant heterogeneity (P < 0.001; I^2^ = 81%). The recurrence-free survival (RFS) was not significantly different between the high and low PD-L1 groups (HR: 1.22; 95% CI 0.97–1.53; P = 0.09) with significant heterogeneity (P < 0.001; I^2^ = 78%). The expression of PD-L1 was found to be significantly correlated with alpha-fetoprotein, hepatitis history, and tumour-infiltrating lymphocytes. Begg’s test found no significant publication bias for OS and RFS. Sensitivity analysis established the robustness of our results. Subgroup analysis and meta-regression analysis found the region of research as a significant contributor to inter-study heterogeneity in RFS, indicating some racial differences in the prognostic role of PD-L1.

**Conclusions:**

Our study found no significant prognostic role of PD-L1 in HCC patients after potential curative hepatectomy based on our included studies. The expression of PD-L1 was significantly correlated with AFP, hepatitis history, and TILs. The prognostic role of PD-L1 in HCC warrants further investigation.

**Electronic supplementary material:**

The online version of this article (10.1186/s12935-019-0738-9) contains supplementary material, which is available to authorized users.

## Background

Hepatocellular carcinoma (HCC) remains one of the most common cancers worldwide. It is ranked as the fifth leading cancer and is the second leading cause of cancer-related mortality [1]. Despite great advances in the past decade, the long-term survival of HCC is low, considering that surgical resection of HCC is only applicable to a small proportion of patients and that the rate of tumour recurrence after surgery of curative intent is high.

The predominant risk factor for HCC in epidemic regions is hepatic inflammatory response following chronic hepatitis B virus (HBV) or hepatitis C virus (HCV) infection [2]. Evidence has suggested that chronic inflammation in the liver creates an immunosuppressive microenvironment that permits HCC tumourigenesis and progression, providing a rationale for targeting the dysregulated tumour microenvironment to improve HCC treatment [3]. Currently, most studies on the mechanisms of HCC immune evasion have focused on the programmed death receptor 1 (PD-1)/programmed death ligand 1 (PD-L1) pathway. PD-1 is an immunoinhibitory receptor expressed in activated T and B cells and natural killer T cells. PD-L1, the major ligand of PD-1, is expressed in various immune cells such as antigen-presenting cells, as well as in endothelial cells. PD-1/PD-L1 ligation suppresses the activation of immune cells as well as the production of certain cytokines such as IFN-γ, thereby inducing immune suppression and peripheral tolerance [4]. Cancer cells hijack this pathway by over-expressing PD-L1 via various mechanisms to protect themselves from the host immune response. Immunotherapies targeting PD-1/PD-L1 immune checkpoints have shown promising efficacy in different malignancies, including HCC [5]. However, the response rate of the PD-1 inhibitor in advanced HCC is far from satisfying [6]. An urgent need exists in the field to further understand the mechanisms of aberrant PD-1/PD-L1 signalling by which HCC forms an immune-evasive tumour microenvironment and for leveraging anti-PD1 immune checkpoint blockade therapies against HCC.

Data on the prognostic role of PD-L1 expression in HCC remain inconsistent. Some studies [7–11] and a meta-analysis [12] have shown that the expression of PD-L1 is correlated with a poor prognosis after hepatectomy, whereas other studies have reported non-homogeneous results [13–15]. To further clarify the prognostic role of PD-L1, the relationship of PD-L1 expression with several important clinicopathological features and tumour-infiltrating T lymphocytes, we conducted this meta-analysis.

## Methods

### Inclusion and exclusion criteria

The inclusion criteria were as follows: (i) study design: cohort studies or case–control studies; (ii) participants: all patients received potential curative hepatectomy for HBV-related HCC; (iii) group: immunohistochemical (IHC) assay of PD-L1 expression in tumour tissues (especially tumour cells); and (iv) sufficient information regarding the role of PD-L1 in recurrence-free survival (RFS) or overall survival (OS) of HCC and/or correlation with clinical features and tumour-infiltrating T lymphocytes (TILs).

The exclusion criteria were as follows: (i) abstracts, letters, editorials, expert opinions, reviews and meta-analyses lacking original data; (ii) studies including patients receiving other curative or palliative therapy (ablation, systemic chemotherapy, radiation, or transarterial chemoembolization) or transplantation; (iii) including data from other cancer types; (iv) other detection methods of PD-L1 expression (non-IHC assays such as ELISA, and/or investigating the expression of PD-L1 in peritumoural tissues or plasma other than tumour tissues).

### Search strategy

PubMed, EMBASE and the Science Citation Index Expanded were searched without language restrictions (the most recent published report search date: December 20, 2018). The search terms used were “hepatocellular carcinoma” and “PD-L1”. The detailed search strategies are described in Additional file 1: Table S1. To identify relevant trials, we also searched the reference lists of related articles. Grey literature (reports and papers that were not published in PubMed, EMBASE and the Science Citation Index Expanded) were not included in this study.

### Data collection and assessment of bias

Two authors (GML and XGL) independently extracted the data concerning the first author’s name, year of publication, country, clinicopathological features of included patients, expression pattern of PD-L1 (sample uses, detection method, cut-off value, positive or high expression rate of PD-L1), and outcomes (survival outcomes and adverse events). For studies including several subgroups, only patients without adjuvant therapy were included. For studies investigating PD-L1 in tumour cells or in immune cells separately, only data from tumour cells were included. When the data were not available in the published reports, additional information was retrieved via correspondence with the primary investigators. Any disagreement between the investigators was resolved via discussion.

### Data pooling and analysis

Pair-wise meta-analyses were conducted using a random-effects models in consideration of inter-study heterogeneity. All analyses were performed using Review Manger 5.3 (Revman, the Cochrane Collaboration) and Stata V.12 software (StataCorp, College Station, Texas, USA) according to the recommendations of the Cochrane Handbook [16] and were reported in line with the preferred reporting items for systematic reviews and meta-analyses (PRISMA) statement [17] (Additional file 2: Table S2). The prognostic role of PD-L1 was expressed as hazard ratios (HRs) and 95% confidence intervals (CIs) and was extracted using a spreadsheet developed by Tierney et al. [18]. ORs with 95% CIs were calculated to assess the correlation of PD-L1 expression with clinicopathological features and TILs. The between-study heterogeneity was assessed using Higgins’ I^2^ statistic. Publication bias was evaluated using funnel plots and Begg’s test. Subgroup analysis and sensitivity analysis by omitting one study in each turn were used to evaluate the reliability of the results. To evaluate the effects of covariates on the pooled estimates and heterogeneity across studies, meta-regression analysis was conducted with covariates including publication year, sample size, positive rate of PD-L1, region of research, proportion of males, proportion of HBV, proportion of single tumours, proportion of vascular invasion and proportion of poor differentiation. P < 0.05 was regarded as significant.

## Results

### Description of the studies

**Fig. 1:**
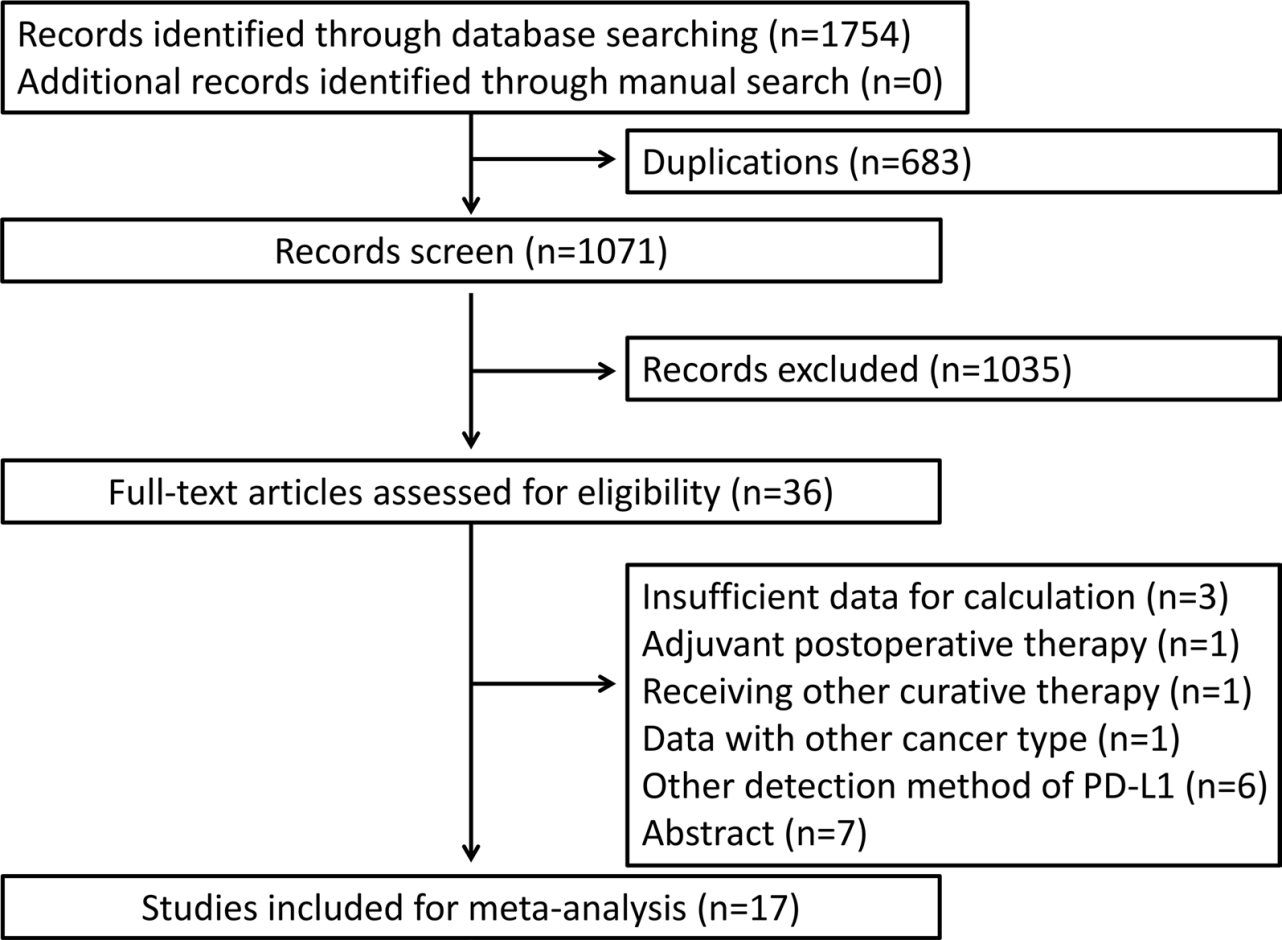
Flow diagram for study selection

The literature searches identified 1754 potentially relevant records (Fig. 1). After excluding 683 duplicated studies, 1071 studies were screened by title and abstract. Thirty-six articles were left for full-text assessment for eligibility. Nineteen records were excluded due to insufficient data (n = 3), including patients receiving adjuvant postoperative therapy (n = 1), including patients receiving other curative or palliative therapy (n = 1), including data from other cancer type (n = 1), employing another detection method of PD-L1 (n = 6) and lacking original data (n = 7). Seventeen studies [7–11, 13–15, 19–27] including 2979 patients were eligible as listed in Table 1. Fifteen studies reported the effect of PD-L1 expression on overall survival [8–11, 13–15, 20–27], while 14 studies reported the effect of PD-L1 expression on recurrence-free survival [7–9, 13, 15, 19–27]. Detailed clinicopathological features and data on TILs are listed in Additional file 3: Table S3. Thirteen studies [7–9, 11, 13, 14, 20–22, 24–27] were conducted in Asia, whereas only four studies [10, 15, 19, 23] were performed in the USA and Europe. PD-L1 expression was characterized as positive or high in 938 patients with a positive ratio ranging from 16.7 to 82.9%.

**Table 1:** Main characteristics of the included studies

Study	Country	Sample size	Stage	Detection method	PD-L1 + n (%)	High PD-L1 cut-off value	Tested samples	Outcome	High PD-L1 prognostic effect
Gao [8]	China	240	I–III	IHC, IPP software	60 (25)	>75%	Tumour tissue sections	RFS; OS	Poorer significant
Wu [11]	China	71	I–IV	IHC, IPP software	35 (49)	> Median	Tumour tissues sections	OS	Poorer significant
Umemoto [21]	Japan	80	I–IV	IHC, intensity of the staining scoring	37 (46)	Score 3	Tumour tissue sections	RFS; OS	Poorer nonsignificant
Kan [14]	China	128	I–IV	IHC,positive cell rate	105 (82)	> 25%	Tumour tissue sections	OS	Favourable significant
Calderaro [19]	France	217	0–C	IHC, positive cell rate	36 (17)	> 1%	Tumour tissue sections	RFS	Poorer nonsignificant
Chen [13]	China	217	I–III	IHC, positive cell rate	52 (24)	> 5%	Tumour tissue sections	RFS, OS	Favourable significant
Gabrielson [23]	USA	58	I–IV	IHC, intensity and distribution	19 (32.8)	NR	Tumour tissue sections	RFS, OS	Favourable nonsignificant
Xie [24]	China	90	I–III	IHC, positive cell rate	15 (16.7)	> 5%	Tumour tissue sections	RFS, OS	Favourable nonsignificant
Chang [7]	Korea	146	T1–T4	IHC, slide scanner for positive cell rate	80 (54.8)	Median	Tumour cell sections	RFS	Poorer significant
Huang [20]	China	411	I–III	IHC, positive cell rate	78 (19)	> 5%;	Liver cancer tissues	RFS, OS	Poorer nonsignificant
Jung 2017 [9]	Korea	85	I–IV; A–D	IHC, staining percentage and intensity scoring	23 (27)	3–5	Tumour cell sections	RFS, OS	Poorer significant
Semaan [10]	Germany	176	T1–T4	IHC, Tissue studio v.2.1 semiquantitative software	88 (50)	Median	All tumour specimens	OS	Poorer significant
Sideras 2017 [15]	Netherlands	146	NR	IHC, staining intensity scoring	121 (82.9)	Cut-off with the lowest-2 log likelihood	Tumour tissue sections	RFS, OS	Favourable significant
Chang [22]	China	145	NR	IHC, positive cell rate	40 (27.6)	> 5%	Whole tissue sections	RFS, OS	Favourable nonsignificant
Dai^1^ [25]	Hong Kong	90	NR	Staining intensity	37 (41.1)	Score 2–3	Tumour tissue sections	RFS, OS	Poorer significant
Dai^2^ [25]	Hong Kong	90	NR	Staining intensity	44 (48.89)	Score 2–3	Tumour tissue sections	RFS, OS	Poorer significant
Hu [26]	China	136	I–III	IHC, positive cell rate of membranous staining	26 (19.1)	> 1%	Tumour tissue sections	RFS, OS	Poorer significant
Liu [27]	China	453	I–KIV	IHC, positive cell rate	87 (19.2)	≥ 5%	Tumour tissue sections	RFS, OS	Poorer significant

Dai et al. [25] reported data from two independent cohort. Dai^1^ represented data from the Prince of Wales Hospital (Hong Kong, China); Dai^2^ represented data from their independent validation cohort as reported

*HR* hazard ratio, *IHC* immunohistochemical assay, *PD-L1* programmed death ligand 1, *RFS* recurrence-free survival, *OS* overall survival

### Prognostic role of PD-L1 expression after hepatectomy for HCC

**Fig. 2:**
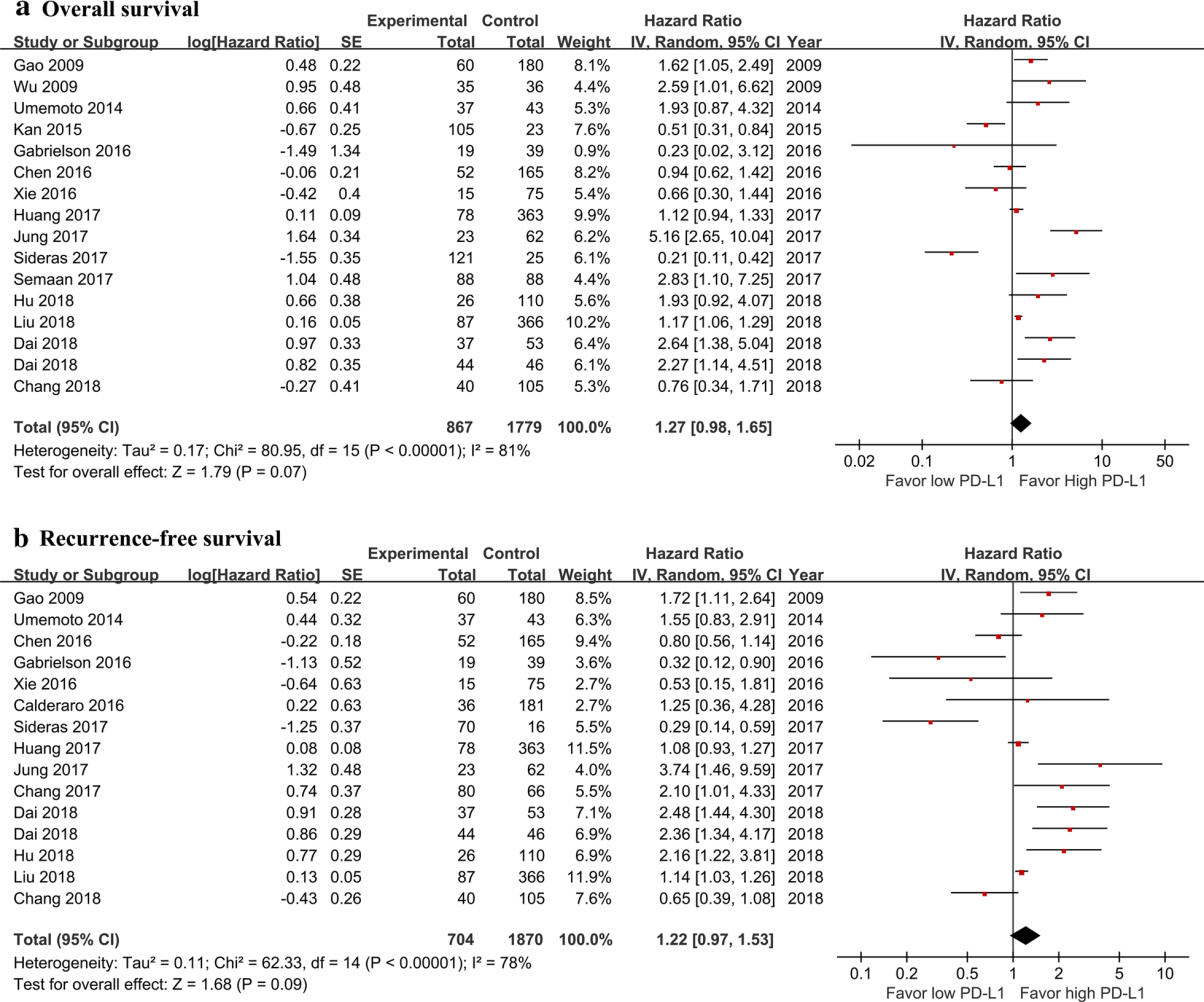
Forest plot for the prognostic role of PD-L1 in overall survival (**a**) and recurrence-free survival (**b**) after potential curative hepatectomy. *CI* confidence interval, *HR* hazard ratio, *PD-L1* programmed death ligand 1

By pooling the data of 15 studies [8–11, 13–15, 20–27], the OS was not found to be significantly different between the high and low PD-L1 groups (HR: 1.27; 95% CI 0.98–1.65; *P *= 0.07) with significant heterogeneity (*P* < 0.001; *I*^2^ = 81%) (Fig. 2a).

By pooling the data of 14 studies [7–9, 13, 15, 19–27], the RFS was not found to be significantly different between the high and low PD-L1 groups (HR: 1.22; 95% CI 0.97–1.53; *P *= 0.09) with significant heterogeneity (*P* < 0.001; *I*^2^ = 78%) (Fig. 2b).

### Correlation between PD-L1 expression and clinicopathological features

**Fig. 3:**
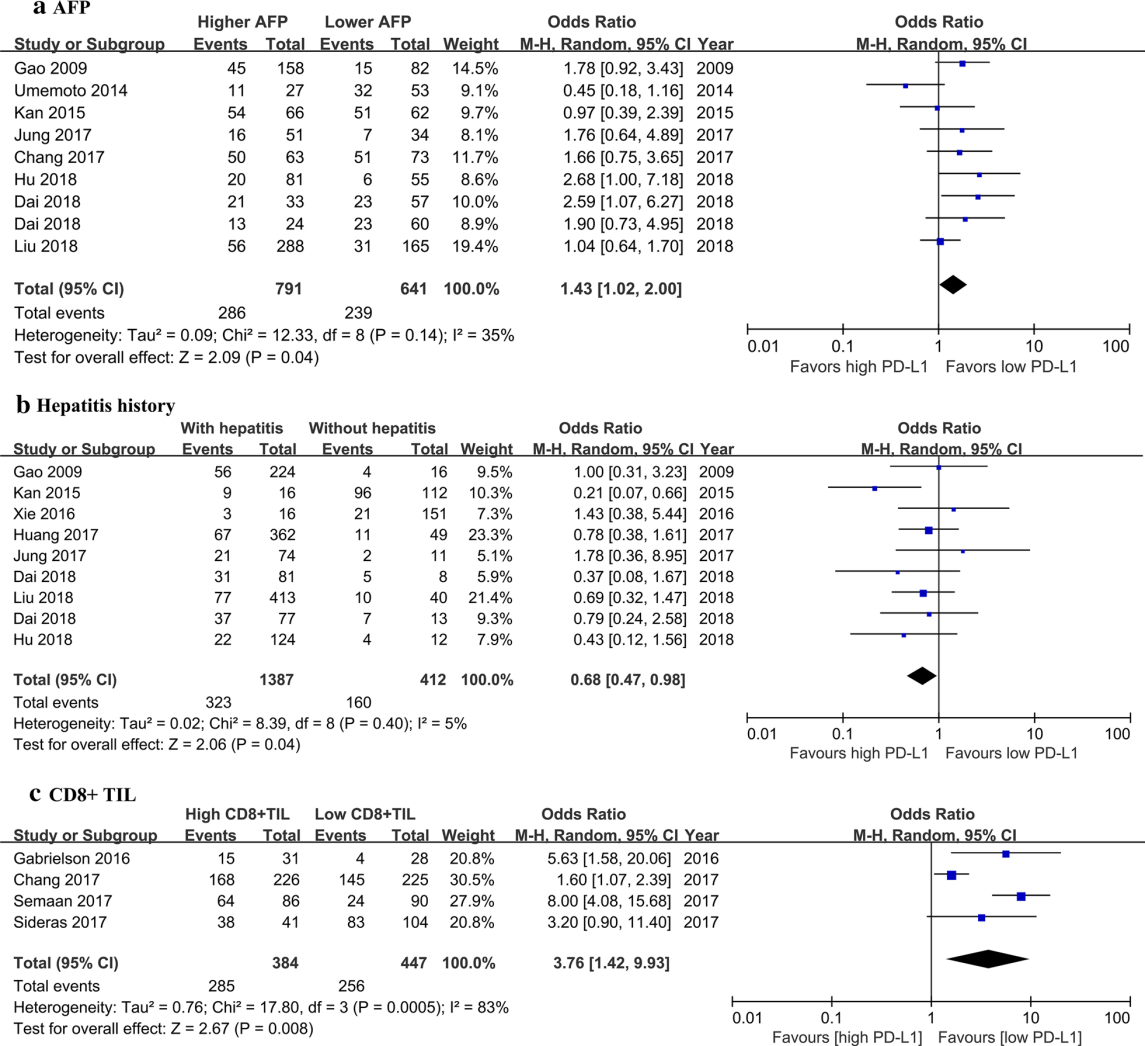
Forest plot for the association of PD-L1 and AFP (**a**), hepatitis history (**b**), and CD8+ TILs (**c**). *AFP* alpha-fetoprotein, *CI* confidence interval, *HR* hazard ratio, *OR* odds ratio, *PD-L1* programmed death ligand 1, *TIL* tumour-infiltrating lymphocyte

We elevated the correlation between PD-L1 expression and clinicopathological features using the OR and 95% CI (Table 2). At least three studies were included in the analyses. The clinicopathological features included age, gender, hepatitis history, AFP, tumour size, tumour stage, tumour differentiation, vascular invasion, tumour multiplicity, encapsulation, and liver cirrhosis. As shown in Fig. 3a, b, alpha-fetoprotein (AFP) and hepatitis history were found to be significantly correlated with the expression of PD-L1 (P = 0.04 and P = 0.04, respectively). No significant association was found between the expression of PD-L1 and other clinicopathological features above.

**Table 2:** Correlation between PD-L1 expression and clinicopathological features

Clinical characteristics	Number of studies	Cases	Odds ratio (95% CI)	P	Heterogeneity	
					I^2^ (%)	P
Gender (male/female)	11	2390	1.09 (0.78, 1.51)	0.62	21	0.24
Age (older/younger)	8	1535	0.93 (0.73, 1.19)	0.58	0	0.92
Hepatitis (yes/no)	8	1799	0.68 (0.47, 0.98)	*0.04*	5	0.40
Cirrhosis (yes/no)	6	999	1.12 (0.74, 1.70)	0.59	30	0.20
AFP (higher/lower)	8	1432	1.43 (1.02, 2.00)	*0.04*	35	0.14
Tumour size (larger/smaller)	8	1688	1.30 (0.88, 1.92)	0.19	55	*0.03*
Tumour encapsulation (no/yes)	4	933	0.91 (0.47, 1.77)	0.79	68	*0.03*
Tumour number (multiple/single)	8	1731	1.05 (0.75, 1.49)	0.77	31	0.18
Vascular invasion (yes/no)	9	2050	1.62 (0.99, 2.64)	0.06	75	< *0.001*
TNM stage (advanced/earlier)	11	2169	1.04 (0.73, 1.50)	0.81	42	0.07

*AFP* alpha-fetoprotein, *CI* confidence interval, *PD-L1* programmed death ligand 1, *TNM* tumour-node-metastasis

The italic P value refers to P < 0.05

### Correlation between PD-L1 expression and TILs

Limited data have shown the correlation between PD-L1 expression and TILs in our included studies. By pooling the data of four studies [7, 10, 15, 23], high PD-L1 expression was correlated with high CD8+ TILs (OR: 3.76; 95% CI 1.42–9.93; P = 0.008) with significant heterogeneity (*P *= 0.0005; *I*^2^ = 83%) (Fig. 3c). Gabrielson et al. reported that PD-L1 expression was correlated with high CD3+ TILs (OR: 3.95; 95% CI 1.20–13.06; P = 0.02). Chang et al. reported that PD-L1 expression was correlated with high PD1+ TILs (OR: 6.89; 95% CI 2.50–19.02; P = 0.0002).

### Publication bias

**Fig. 4:**
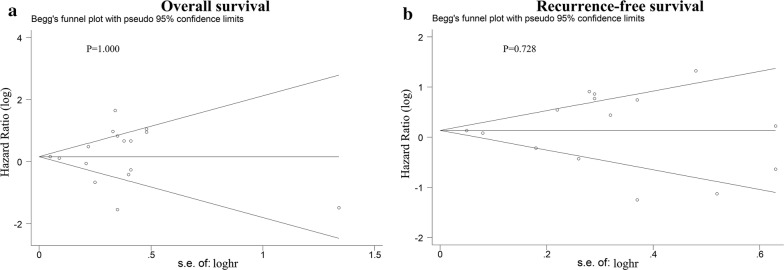
Begg’s funnel plot for publication bias tests in **a** overall survival (OS) and **b** recurrence-free survival (RFS) in HCC. *CI* confidence interval, *HR* hazard ratio, *HCC* hepatocellular carcinoma

Begg’s funnel plot was used to evaluate publication bias. As shown in Fig. 4a, b, the results showed no publication bias for OS and RFS, with P values of 1.000 and 0.728, respectively. Publication bias was not analysed for the correlation of PD-L1 expression and clinicopathological features or TILs because the number of included studies was less than 10 in most groups due to the low sensitivity of the qualitative and quantitative tests [28].

### Subgroup analyses and sensitivity analysis

**Fig. 5:**
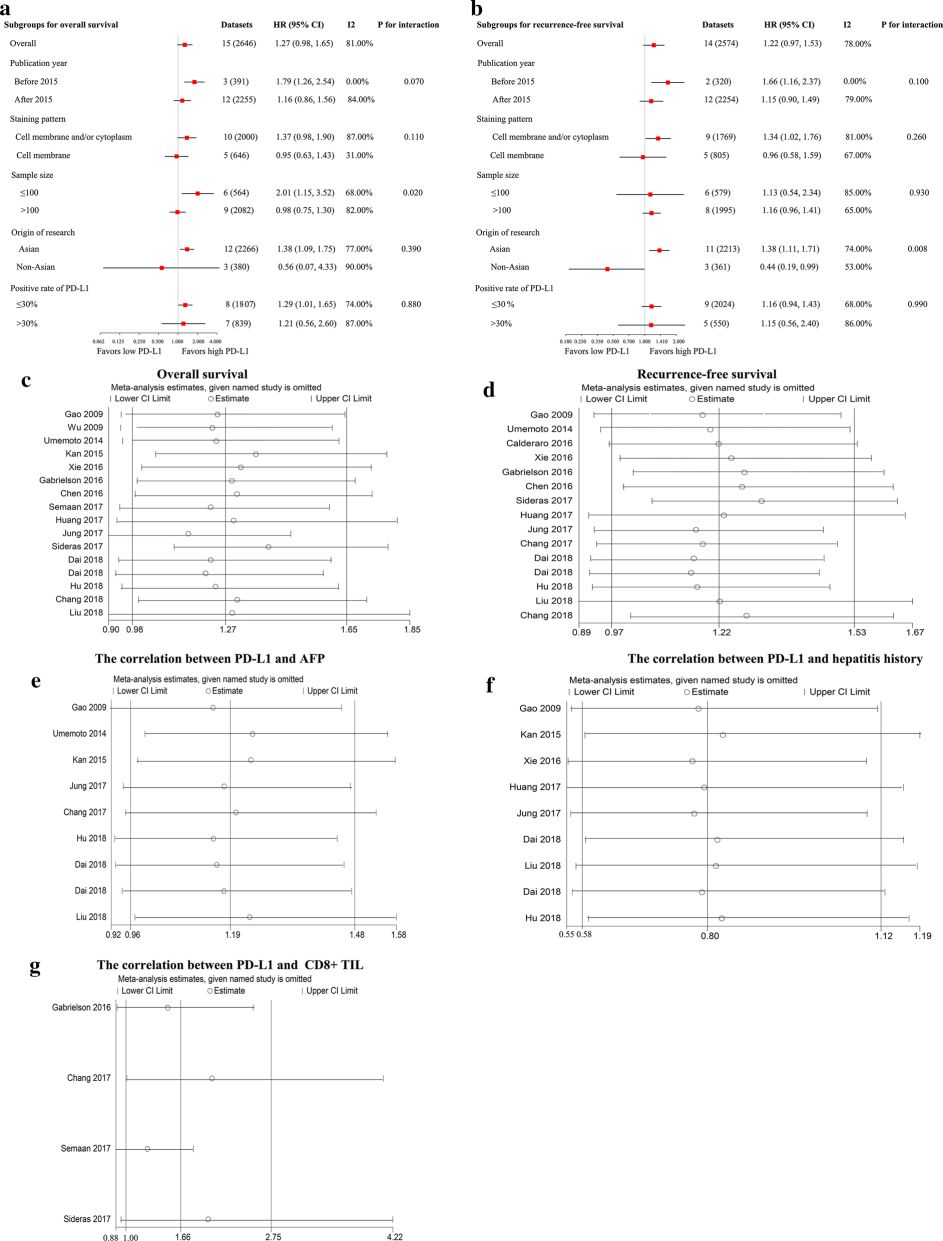
Results of subgroup analysis and sensitivity analysis. Forest plot of the subgroup analysis according to publication year (before 2015 and after 2015), origin of research (Asian and non-Asian), sample size (> 100 and < 100), and PD-L1 positive or high expression rate (< 30% and ≥ 30%) for the prognostic role of PD-L1 in **a** overall survival (OS) and **b** recurrence-free survival (RFS) in HCC. Sensitivity analysis revealed that no study exerted a significant influence on the overall pooling result of OS (**c**) and RFS (**d**). Sensitivity analysis revealed that no study exerted a significant influence on the overall pooling correlation of PD-L1 and AFP (**e**), hepatitis history (**f**), and CD8+ TILs (**g**). *AFP* alpha-fetoprotein, *CI* confidence interval, *HR* hazard ratio, *OR* odds ratio, *PD-L1* programmed death ligand 1, *HCC* hepatocellular carcinoma, *TIL* tumour-infiltrating lymphocyte

We conducted subgroup analyses according to publication year (before 2015 and after 2015), the origin of research (Asian and non-Asian), sample size (> 100 and < 100) and rate of positive or high PD-L1 (≤ 30% and > 30%). As shown in Fig. 5a, high PD-L1 was significantly correlated with poorer OS when combing data published before 2015, or with a sample size smaller than 100, or data from Asian populations, or studies reporting ≤ 30% of positive PD-L1. As shown in Fig. 5b, high PD-L1 was significantly correlated with poorer RFS when combining data published before 2015, or a cell membrane or cytoplasm PD-L1 staining pattern. Particularly, a significant difference was found in the prognostic role of PD-L1 between data from the Asian and non-Asian subgroups (P = 0.008). In the Asian subgroup, high PD-L1 indicated a significantly poor RFS (HR: 1.38; 95% CI 1.11–1.71; P = 0.003). However, in the non-Asian subgroup, high PD-L1 indicated an almost but not significant better RFS (HR: 0.44; 95% CI 0.19–0.99; P = 0.05). Additionally, the between-study heterogeneity was decreased to some degree in some subgroups. To further examine the robustness of the prognostic role of PD-L1 by sensitivity analyses, we applied a random effects model, omitting one study in each turn. No study exerted a significant influence on the overall pooling result, indicating that our estimates were robust and reliable (Fig. 5c–g, Additional file 4: Figure S1).

### Meta-regression analysis

**Fig. 6:**
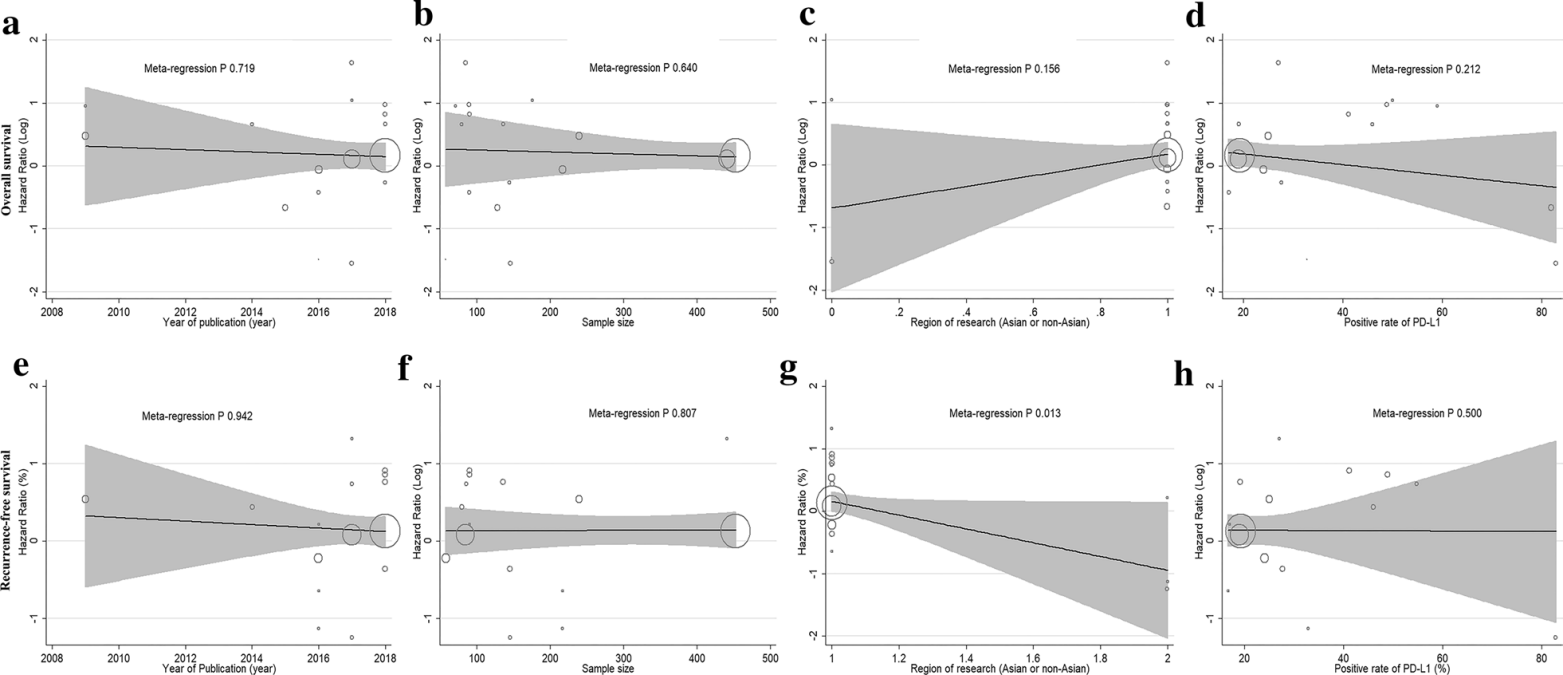
Meta-regression analysis for overall survival and recurrence-free survival in HCC. Bubble plot with a fitted meta-regression line of the log hazard ratio for **a**, **e** publication year, **b**, **f** sample size, **c**, **g** region of research, and **d**, **h** positive rate of PD-L1 (%). **a**–**d** Represent overall survival; **e**–**h** represent recurrence-free survival. The size of circles is proportional to the weight of each study in the fitted random-effects meta-regression. *PD-L1* programmed death ligand 1, *HCC* hepatocellular carcinoma

For OS, meta-regression analysis showed a trend for a positive rate of PD-L1, region of research, proportion of male, proportion of HBV, proportion of single tumours, proportion of vascular invasion and proportion of poor differentiation, but the trend was not statistically significant (all P > 0.05, Fig. 6a–d, Additional file 5: Table S4). Given the significant heterogeneity in the overall effect estimates, the contribution of different study characteristics to the level of heterogeneity was calculated (Additional file 5: Table S4). No significant factors contributed to the level of heterogeneity, and the proportion of heterogeneity ranged from − 8.73% to 11.16% (all P > 0.05). The remaining heterogeneity was large (τ^2^ range from 0.187 to 0.628). For RFS, meta-regression analysis found that the region of research could explain 51.86% of the between-study variance (Fig. 6g, Additional file 5: Table S4, P 0.013). No other significant factors contributed to the level of heterogeneity, and the proportion of heterogeneity ranged from − 13.61% to 15.06% (all P > 0.05); the remaining heterogeneity was large (τ^2^ range from 0.172 to 0.407) (Fig. 6e, f, h; Additional file 5: Table S4). The meta-regression analysis categorized by age, AFP, proportion of cirrhosis, proportion of larger tumour size, proportion of tumour encapsulation, and TNM stage was not achieved and was attributed to the lack of data in the included studies.

## Discussion

While numerous studies have established that aberrant expression of PD-L1 in cancer promoted cancer immune escape and blockade of the PD-L1-enhanced anti-tumour response [4, 5], the prognostic role of PD-L1 in HCC has remained inconsistent. Several previous studies [8, 11, 21] and meta-analyses [29–31] have shown that high PD-L1 indicates a poorer OS and RFS, which could be explained by innate immune resistance. The innate immune resistance implies the constitutive expression of PD-L1, which is driven by intrinsic cellular oncological signalling in tumour cells and can lead to tumour-infiltrating immune cell inhibition [32, 33]. In this sense, PD-L1 expression reflects a lack of anti-tumour immunity and is reasonable to correlate with a significantly poorer prognosis [7–11, 21]. Meanwhile, a few recent studies failed to confirm the negative prognostic effect of PD-L1 and even showed the opposite [13–15, 23, 24]. For instance, Sideras et al. [15] reported that high PD-L1 in tumour cells indicated a significant better survival in HCC. The adaptive immune resistance was used to explain the inconsistency. The adaptive immune resistance implied a specific state of immune privilege of tumour cells acquired by the induced expression of PD-L1 to inhibit the pre-existing anti-tumour T cell response. The state can be reversed easily by blocking the PD1/PD-L1 interaction [34, 35]. From this point of view, the upregulation of PD-L1 represents the existence of immune-surveillance and could be correlated with a better prognosis [13–15]. Both mechanisms may co-exist, and the predominant mechanism may shift from one to the other at different times depending on the cancer immunogenicity [36]. The prognostic role of PD-L1 tends to be the combined effect of these two mechanisms. In the present meta-analysis based on 17 studies with 2979 patients, we found no significant prognostic role of PD-L1 in HCC after potential curative hepatectomy. Additionally, the expression of PD-L1 was correlated with AFP, hepatitis history, and CD8+ TILs but not with other clinicopathological features. The robustness and reliability of these results was further validated by sensitivity analysis.

Considering the significant between-study heterogeneity, we conducted further subgroup analysis and meta-regression analysis. High PD-L1 predicted a poorer RFS in Asian studies, while high PD-L1 predicted a better RFS in non-Asian patients; the difference between the subgroups was significant, and meta-regression analysis further confirmed the results. Together, these results indicated some racial differences in the prognostic role of PD-L1. Theoretically, several factors might also help explain the great heterogeneity. First, the positive rate of PD-L1 varied greatly across the included studies due to the different tested tissue samples, IHC assay, detection antibody, staining pattern, analytic method, and cut-off value. For instance, PD-L1 can be expressed in both tumour cells and immune cells in the intra-tumoural area of HCC tissues, with different biological and prognostic roles. As Liu et al. [27] reported, PD-L1 expressed on tumour cells was negatively correlated with the prognosis, while PD-L1 expressed on macrophages was positively correlated with the prognosis in HCC. Some included studies investigated the overall expression in the intra-tumoural area [10] or simply classified PD-L1 expression on tumour cells or immune cells by IHC [19]. Additionally, by combining the data published before 2015 or data including PD-L1 staining both in the cell membrane and/or cytoplasm, we found high PD-L1 significantly predicted a poorer survival. However, earlier studies might have used an invalid PD-L1 antibody [8, 11], while more recent studies utilized an antibody that distinguished between cytoplasmic and membranous patterns of PD-L1 staining [9, 15, 20, 22]. As a type I transmembrane molecule [37], membranous PD-L1 was proposed to be the most functionally relevant, and cytoplasmic staining represents intracellular stores of PD-L1 ready to be transported to the cell membrane with appropriate stimulation [38]. Failing to distinguish the expression pattern of PD-L1 in HCC can potentially confound the positive rate of PD-L1 and prognostic role of HCC. Moreover, the analytic method varied extensively. Some studies have utilized imaging software [7, 8, 10, 11, 13, 14, 19, 20, 22, 24], while others were based on intensity and distribution [9, 15, 21, 23]. Finally, the cut-off value used to evaluate high and low PD-L1 expression varied from 1 to 75%. Second, the different baseline characteristics of included patients and different follow-up periods might be another contributing factor. For example, the study of Calderaro et al. [19] only evaluated the prognostic role of PD-L1 in early recurrence within 2 years, while some other studies performed a significant longer follow up (both longer than 80 months) [21, 22]. However, our meta-regression analysis did not find a factor other than the region of research that significantly contributed to the level of heterogeneity, and the roles of the abovementioned factors warrant further investigation. Further studies should make great efforts to establish standardization regarding the methodology of PD-L1 assessment, such as the method to distinguish between tumour cells and immune cells, to distinguish between the expression patterns of PD-L1, to accurately calculate PD-L1 staining cells, and to set optimal cut-off values. Additionally, critical baseline patient characteristics such as AFP must be considered when estimating the prognostic role of PD-L1.

There have been several meta-analyses to date [12, 29, 31, 39–43]. Most recently, Li et al. [43] finished a similar meta-analysis on the value of PD-L1 in HCC patients. The major difference between our study might be the included criteria. Li et al. included all studies that examined PD-L1 expression levels in clinical HCC tissues. They included the study from Zeng et al. [44] that evaluated most of the PD-L1 expression using flow cytometry, but not IHC, and the study from Dai et al. [45] that assessed the PD-L1 expression in peritumoural tissues. Additionally, they included the study from Finkelmeier et al. [46] that investigated soluble PD-L1 in serum. As pointed out in a previous study, using data from different assays or different tissue types would inevitably raise the heterogeneity more or less [12]. Therefore, our meta-analysis focused on only the immunohistochemical (IHC) assay of PD-L1 expression in tumour tissues (or tumour cells but not immune cells in the intra-tumoural area) and found some inconsistent results with previous meta-analyses. Although our results should be explained with great caution, our study possessed some strengths. To our knowledge, the present study is the largest meta-analysis to date with subgroup and meta-regression analyses providing extended evidence regarding the inter-study heterogeneity issues.

Several limitations existed in our study. First, most of the included studies were retrospective and of questionable quality, and data from the prospective high-quality study were lacking. Second, significant between-study heterogeneity was found. Nevertheless, we conducted these meta-analyses using random-effect models, and sensitivity analysis confirmed the reliability of our results. Third, we could not evaluate the prognostic role of PD-L1 based on critical prognostic factors such as TNM stage or HCC causes because most studies did not report sufficient data. For example, as Huang et al. [20] reported, high PD-L1 expression was correlated with a high HBV viral load. How the causes of HCC, such as hepatitis C virus or alcohol, influence the expression and prognostic role of PD-L1 remain unclear. Finally, our analysis indicated some racial differences exist in the prognostic role of PD-L1. However, data from non-Asian patients were limited, and the prognostic role of PD-L1 in these patients warrants further investigation.

## Conclusions

Despite the limitations listed above, our meta-analyses found no significant prognostic role of PD-L1 in HCC patients after potential curative hepatectomy based on the included studies. The expression of PD-L1 was significantly correlated with AFP, hepatitis history, and TIL. The prognostic role of PD-L1 in HCC warrants further investigation.

## Additional files

**Additional file 1: Table S1.** Search strategy.

**Additional file 2: Table S2.** Preferred Reporting Items for Systematic reviews and Meta-Analyses (PRISMA) checklist.

**Additional file 3: Table S3.** Association of PD-L1 and clinicopathological features and tumour-infiltrating lymphocytes of the included studies.

**Additional file 4: Figure S1.** Results of sensitivity analysis for the correlation of PD-L1 and several clinicopathological features. Sensitivity analysis revealed that no study exerted a significant influence on the overall pooling correlation of PD-L1 and gender (A), tumour differentiation (B), tumour stage (C), vascular invasion (D), tumour size (E), age (F), tumour multiplicity (G), cirrhosis (H), and tumour encapsulation (I). PD-L1: programmed death ligand 1; HCC, hepatocellular carcinoma.

**Additional file 5: Table S4.** Meta-regression analysis for overall survival and recurrence-free survival.

## Abbreviations

AFP: alpha-fetoprotein
CI: confidence interval
HBV: hepatitis B virus
HCC: hepatocellular carcinoma
HR: hazard ratio
IHC: immunohistochemical
OS: overall survival
OR: odds ratio
PD-L1: programmed death ligand 1
PD-1: programmed death receptor 1
RFS: recurrence-free survival
TNM: tumour-node-metastasis
TIL: tumour-infiltrating T lymphocyte
