# Supplementary material for: Prognostic role of PD-L1 for HCC patients after potentially curative resection: a meta-analysis
Source: Cancer Cell Int. 2019 Jan 29;19:22. doi: 10.1186/s12935-019-0738-9 (PMC6352338; doi:10.1186/s12935-019-0738-9)
Supplement: Supplementary file 1 — Additional file 1: Table S1. Search strategy. [file 12935_2019_738_MOESM1_ESM.doc]

**Table S1.** Search strategy

| **MEDLINE (PubMed),** from dates of inceptions up to December 20, 2018 |
| --- |
| #1 "Liver Neoplasms"[Mesh] OR "Carcinoma, Hepatocellular"[Mesh]  #2 ((((liver OR hepatic OR hepatocellular OR hepato-cellular) AND (cancer* OR carcinom* OR neoplasm? OR maglinan* OR tumor* OR tumour*))) OR (HCC OR hepatoma？))  #3 #1 OR #2  #4 "CD274 protein, human"[Supplementary Concept]  #5 PDCD1LG1 protein or programmed cell death 1 ligand 1 protein or CD274 antigen or B7-H1 or B7H1 or PD-L1 or PDL1  #6 #4 OR # 5  #7 #3 AND #6 |
| **EMBASE,** from dates of inceptions up to December 20, 2018 |
| #1 'liver cell carcinoma'/exp  #2 (liver:ti,ab OR hepatic:ti,ab OR hepatocellular:ti,ab OR 'hepato cellular':ti,ab) AND [embase]/lim  #3 (cancer*:ab,ti OR carcinom*:ab,ti OR neoplasm?:ab,ti OR maglinan*:ab,ti OR tumor*:ab,ti OR tumour*:ab,ti) AND [embase]/lim  #4 #2 AND #3  #5 (hcc:ab,ti OR hepatoma:ab,ti) AND [embase]/lim  #6 #1 OR #4 OR #5  #7 'programmed death 1 ligand 1'/exp  #8 (((pdcd1lg1:ab,ti AND protein:ab,ti OR programmed:ab,ti) AND cell:ab,ti AND death:ab,ti AND ligand:ab,ti AND 1:ab,ti AND protein:ab,ti OR cd274:ab,ti) AND antigen:ab,ti OR 'b7 h1':ab,ti OR b7h1:ab,ti OR 'pd l1':ab,ti OR pdl1:ab,ti) AND [embase]/lim  #9 #7 OR #8  #10 #6 AND #9 |
| **Science Citation Index Expanded,** from dates of inceptions up to December 20, 2018 |
| #1 TS= ((((liver OR hepatic OR hepatocellular OR hepato-cellular) AND (cancer* OR carcinom*OR neoplasm? OR maglinan* OR tumor* OR tumour*))) OR (HCC OR hepatoma？))  #2 TS= PDCD1LG1 protein or programmed cell death 1 ligand 1 protein or CD274 antigen or B7-H1 or B7H1 or PD-L1 or PDL1  #3 #1 AND #2 |
