# Supplementary figures and images for: Prognostic role of PD-L1 for HCC patients after potentially curative resection: a meta-analysis
Source: Cancer Cell Int. 2019 Jan 29;19:22. doi: 10.1186/s12935-019-0738-9 (PMC6352338; doi:10.1186/s12935-019-0738-9)

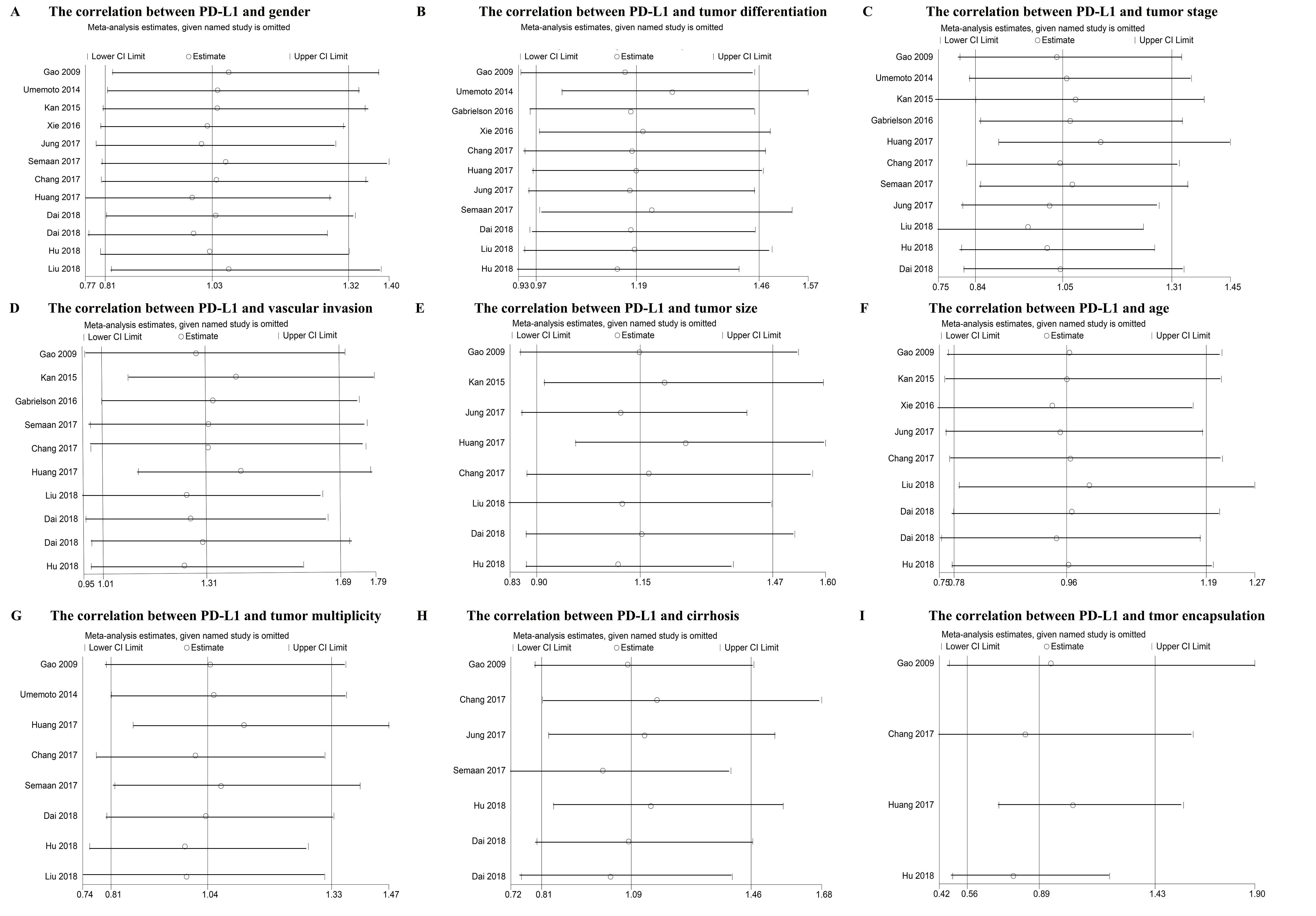

Supplement: Supplementary file 4 — Additional file 4: Figure S1. Results of sensitivity analysis for the correlation of PD-L1 and several clinicopathological features. Sensitivity analysis revealed that no study exerted a significant influence on the overall pooling correlation of PD-L1 and gender (A), tumour differentiation (B), tumour stage (C), vascular invasion (D), tumour size (E), age (F), tumour multiplicity (G), cirrhosis (H), and tumour encapsulation (I). PD-L1: programmed death ligand 1; HCC, hepatocellular carcinoma. [file 12935_2019_738_MOESM4_ESM.tif]
